# Variation in Point-of-Care Testing of HbA1c in Diabetes Care in General Practice

**DOI:** 10.3390/ijerph14111363

**Published:** 2017-11-09

**Authors:** Troels Kristensen, Frans Boch Waldorff, Jørgen Nexøe, Christian Volmar Skovsgaard, Kim Rose Olsen

**Affiliations:** 1 COHERE, Department of Public Health & Research Unit of General Practice, University of Southern Denmark, 5000 Odense C, Denmark; krolsen@sam.sdu.dk; 2 Research Unit of General Practice, University of Southern Denmark, 5000 Odense C, Denmark; fwaldorff@health.sdu.dk (F.B.W.); jnexoe@health.sdu.dk (J.N.); 3 COHERE, Department of Business and Economics, University of Southern Denmark, 5230 Odense M, Denmark; chsko@sam.sdu.dk

**Keywords:** point-of-care testing, diabetes, general practice, HbA1c, variation, incentives, organization, patient data, family medicine, management

## Abstract

*Background:* Point-of-care testing (POCT) of HbA1c may result in improved diabetic control, better patient outcomes, and enhanced clinical efficiency with fewer patient visits and subsequent reductions in costs. In 2008, the Danish regulators created a framework agreement regarding a new fee-for-service fee for the remuneration of POCT of HbA1c in general practice. According to secondary research, only the Capital Region of Denmark has allowed GPs to use this new incentive for POCT. The aim of this study is to use patient data to characterize patients with diabetes who have received POCT of HbA1c and analyze the variation in the use of POCT of HbA1c among patients with diabetes in Danish general practice. *Methods:* We use register data from the Danish Drug Register, the Danish Health Service Register and the National Patient Register from the year 2011 to define a population of 44,981 patients with diabetes (type 1 and type 2 but not patients with gestational diabetes) from the Capital Region. The POCT fee is used to measure the amount of POCT of HbA1c among patients with diabetes. Next, we apply descriptive statistics and multilevel logistic regression to analyze variation in the prevalence of POCT at the patient and clinic level. We include patient characteristics such as gender, age, socioeconomic markers, health care utilization, case mix markers, and municipality classifications. *Results:* The proportion of patients who received POCT was 14.1% and the proportion of clinics which were “POCT clinics” was 26.9%. There were variations in the use of POCT across clinics and patients. A part of the described variation can be explained by patient characteristics. Male gender, age differences (older age), short education, and other ethnicity imply significantly higher odds for POCT. High patient costs in general practice and other parts of primary care also imply higher odds for POCT. In contrast, high patient costs for drugs and/or morbidity in terms of the Charlson Comorbidity index mean lower odds for POCT. The frequency of patients with diabetes per 1000 patients was larger in POCT clinics than Non-POCT clinics. A total of 22.5% of the unexplained variability was related to GP clinics. *Conclusions:* This study demonstrates variation in the use of POCT which can be explained by patient characteristics such as demographic, socioeconomic, and case mix markers. However, it appears relevant to reassess the system for POCT. Further studies are warranted in order to assess the impacts of POCT of HbA1c on health care outcomes.

## 1. Introduction

An important part of managing patients with diabetes relates to efforts focused on controlling hyperglycaemia [1]. Hyperglycaemia is measured using a haemoglobin A1c (HbA1c) test, which assesses the average glucose level of the previous 60–120 days. Near normal glycemic control levels in diabetes care reduce the development and progression of complications [2]. Therefore, it is important to public health that the timely and appropriate management of HbA1c is part of the care pathway for patients with diabetes in primary care [3]. General practitioners often play an important role in the management of HbA1c levels [4].

Point-of-care testing (POCT) denotes any diagnostic test executed at or near the location of the patient [5]. The advantage of POCT is that the near-immediate test results allow patients and doctors to evaluate progress, review results, and establish treatment plans within a single visit. Thus, POCT may improve compliance, diabetes control, and patient outcomes and enhance clinical efficiency with fewer patient visits; subsequently, increasing patient satisfaction and reducing costs [6].

Nevertheless, the distribution and use of POCT in Europe has been found to differ greatly between institutions and countries [7]. One challenge is that the variation in use of POCT may be due to variation in provider characteristics, such as local medical opinions and the allocation of medical resources, rather than patient characteristics such as actual illness or patient preferences [8,9,10]. This study describes variation in POCT of HbA1c in general practice at the patient and clinic levels and analyses the importance of patient level characteristics for the likelihood of receiving POCT of HbA1c.

Recent examinations about the quality of diabetes care have shown a large variation in diabetes care within Denmark [11,12]. The variation in the way HbA1c testing is performed in general practice does not always reflect variation in quality [13]. A part of the reason behind the variation in HbA1c testing may be related to differences in patient characteristics and the way HbA1c testing is organized and incentivized by providers [14]. For instance, general practitioners may perceive POCT of HbA1c to be more relevant for selected patient groups. Other specific reasons may be differences in the politically and locally determined implementation of incentives such as fee-for-service (FFS) and the way general practitioners (GPs) perceive the attractiveness of POCT and these fees [15]. However, very little has been published on variation in this field, even though POCT in diabetes management may benefit patients and reduce costs [6]. To the best of our knowledge, no published studies have investigated variation in the use of POCT of HbA1c in general practice.

Opponents to the present organisation of POCT may argue that the lack of sufficient strength of the way in which the Danish regulators have implemented a new FFS fee for POCT of HbA1c (only in one region) has contributed to variation in the testing of HbA1c in general practice, rather than equal access to POCT [14]. In particular, this type of variation may be unwarranted if the quality of the testing features of POCT technology is equivalent to those performed in a central laboratory [6]. The aim of this paper is to describe and analyse the variation in the use of POCT for HbA1c among patients with diabetes in Danish general practice. First, we describe variation in POCT of HbA1c at patient and clinic levels. Second, we use a set of variables at the individual level such as socioeconomic markers, case mix markers, health care utilizations markers, and a rural classification system to analyse and explain variation in POCT of HbA1c among patients with diabetes who received diabetes care in clinics that were able to perform POCT.

### Organisation of POCT of HbA1c and Incentives for Testing HbA1c in General Practice

The agreement regarding the organization and remuneration of POCT of HbA1c in general practice is an aspect of the contract between the organization of Danish Regions and the General Practitioners Organisation (PLO). Two central features of this contract are politically negotiated fee-for-services fees (70%) and capitation payments (30%), which are paid by the regions since GP services are free of charge for patients [16]. In each region, the local PLO and regional regulators have created a laboratory assurance subcommittee under the national laboratory committee. The purpose of this subcommittee is to facilitate accurate, updated, and improved quality in the area of diagnostics, as well as treatment and analytic work such as decisions regarding the implementation of POCT. Danish guidelines recommend measuring HbA1c levels every three-to-six months for patients with type 2 diabetes. In 2008, a framework agreement was made between PLO and the Board for Wages and Tariffs of the Regions (RNTL) to implement a national FFS fee for POCT of HbA1c. It was agreed that general practitioners are paid a FFS fee of (Danish Krone 115.97 or €15.49) per POCT of HbA1c (October, 2016 prices). Each of the five Danish regions can decide whether to allow their GPs to use this new tariff as an alternative to the fee for standard laboratory testing of blood samples and as an incentive for using POCT in order to promote timely and appropriate diabetes management. In combination with the existing FFS remuneration structure, the new tariff means that GPs can be rewarded for performing HbA1c testing during ordinary control visits in several ways:

(a) Through a standard consultation (Danish Krone 137.83 or €18.48) combined with a blood sample fee (Danish Krone 47.23 or €6.33) for external laboratory testing and a follow-up GP visit or dialog via either mail or telephone (in the case of walk-in laboratory facilities at local laboratories the GP will not receive a fee for laboratory testing).
(b) A standard consultation (Danish Krone 137.83 or €18.48) combined with a fee for POCT of HbA1c (Danish Krone 115.97 or €15.49).

If the GP performs the POCT, they have to deduct the variable cost for cuvettes (approximately €3) and the cost related to depreciation, capital invested in equipment and cost for maintenance, calibration of equipment, and training from the fee. However, POCT equipment only represents a relatively small investment and is often provided free of charge by suppliers. Costs of HbA1c testing equipment in central laboratories are covered by the region.

In addition to HbA1c testing during ordinary control visits via POCT or laboratory testing, typical management of patients with diabetes includes an annual diabetes control visit (Danish Krone 371.09 or €49.74) which rewards the GPs with an FFS fee for the control of chronic diseases [15]. GPs are only allowed to use this annual fee once a year per chronic condition per patient in addition to laboratory and supplementary FFS fees. During a trial period from 2007 to 2014, an alternative annual special fee which included payments for subsequent control consultations could also be used rather than the fee for annual control and related fees for the subsequent one to three control visits [15]. This additional incentive in combination with (a), (b), and the rules regarding the annual fee may have contributed to making the incentives more difficult to understand and created more variation in the applied ways of controlling hyperglycaemia. The fee structure may also have encouraged some GPs to use the fee for POCT of HbA1c in combination with blood samples from external laboratories rather than only as a substitute for testing at external laboratories. However, the fact that the POCT of HbA1c tariff might influence the GP behavior in this direction is not well documented and it is only assessed to be one element behind the variation in implementation. Other elements are patient demand, professional opinions, and attitudes towards POCT of HbA1c.

## 2. Materials and Methods

### 2.1. Data

Register Data from the Danish Drug Register, the Danish Health Service Register, and the National Patient Register (from Statistics Denmark) was used in an algorithm to define a national population of 172,906 patients with diabetes. Danish health care register data covers the entire population and has full coverage. We use a validated algorithm similar to what is used in other studies and by WHO [9,17]. Patients were required to be above 18 years of age, alive, and living in Denmark by 2011. In addition, at least one out of the three following criteria had to be met: (1) The patients have redeemed at least one prescription for anti-diabetic drugs with ATC code A10A* (*: Including subgroups) or/and A10B* in 2011. ATC-code A10BA02 is excluded for women between the ages of 20 to 40 (gestational diabetes); (2) The patients have received at least three blood sugar or HbA1c tests in 2011 (either from their general practitioner or a specialist in the primary care sector); and (3) The patients is registered with one of the following ICD10 codes in the National patient Register in 2011: DE10, DE11, DE12, DE13, DE14, DO24, and DH360. This study focuses on the subgroup of 44,981 patients with diabetes in the Capital Region. Sample availability: Data is protected by Statistics Denmark. Application to access the data must be made to Statistics Denmark in collaboration with a Danish research institution.

### 2.2. Descriptive Statistics

#### 2.2.1. Patient Level

The fee for POCT of HbA1c registered in the Danish Health Service Register was used to construct a dichotomous proxy variable for patients who received either one or more POCT or no POCT. This fee is the best available measure of POCT activity and allowed us to construct descriptive statistics of patients monitored with POCT versus patients controlled without POCT. All patients were described for the following set of markers and characteristics: demographic markers, socioeconomic markers, health care utilization, case mix markers, and a municipality type classification; which are all described below. Next, the differences in mean values, proportions, and distributions of each marker across the POCT and non-POCT groups were tested through unpaired two-sided *t*-tests, the Mann Whitney test, and chi-square-tests, respectively.

The demographic markers included individual level variables on age and gender. The socioeconomic markers comprised data on income, dummy variables on unemployment status, education (only compulsory public school), ethnicity (other than Danish), cohabitation status (single), and retirement status. Health care utilization was described using hospital admissions data for Ambulatory Care Sensitive Conditions (ACSCs), standard hospital admissions, emergency department visits, the number of GP visits, and the number of POCT of HbA1c in general practice. ACSCs are conditions where proper outpatient care can potentially prevent or decrease the need for unnecessary hospitalization; furthermore, early intervention can prevent complications or more severe disease(s) [18,19]. Morbidity burden and patients’ need for health care were measured via the Charlson Comorbidity index, as well as measures of cost of care in primary care and secondary care and drug costs. The Charlson Comorbidity index and inpatient Diagnosis Related Groups (DRG)-costs were used to measure case mix in secondary care [20]. Costs for the remuneration of GP-fees in general practice, other primary care costs, and outpatient hospital-costs in the Danish Ambulatory Grouping System (DAGS) were used as a proxy measure for case mix in primary care and for outpatient case mix for the hospital sector. The justification is that morbidity and primary care costs are linked [21]. Municipality types were portrayed through a rural district classification [22]. This Municipality classification was obtained by dividing the municipalities into four categories: rural, peripheral, intermediate, and urban municipalities.

#### 2.2.2. Clinic Level

The data at individual level was aggregated to measure the prevalence and variation in POCT of HbA1c at the clinic level. Clinics with more than five POCT per year were included and defined as POCT clinics. To describe and compare clinic variation across POCT vs. non-POCT, the following clinic characteristics were used; list size, number of patients with diabetes seen per clinic, number of patients with diabetes per 1000 patients, and the clinic’s municipality type.

### 2.3. Regression Analysis and Data

Due to the binary nature of the POCT event and clustering of patients in GP clinics, we applied a multilevel logistic regression model for binary responses acknowledging clustering of patients within GP clinics to explore the association between POCT and patient characteristics [23]. The analysis was performed on patients with diabetes that were linked to clinics with POCT of HbA1c services because they were the only patients who had a chance of receiving POCT. The latent variable representation of the applied random intercept logistic regression model takes the following form:

(1) $$POCT_{ij}^{*}=\beta_{00}+\beta^{\prime }x_{ij}+\;u_{j\;}+\;\varepsilon_{ij}$$

where the dependent dummy variable *POCT_ij_* was defined by

(2) $$POCT_{ij}=\;\{\begin{array}{c} \;1\;if\;POCT\;\geq 1\\ 0\;\text{otherwise} \end{array}$$

The parameter $x_{ij}$ in (1) is a row vector of explanatory variables containing characteristics of patients i = 1…n in clinic *j*. The term *u_j_* is the random effect of being in group j where *u_j_* ∼N(0,σ_u_^2^). *β* represents within group change. This model allows the probability to vary from clinic to clinic and *ε_ij_* is the residual at the patient level. The total residual variance is Var(POCT_ij_^*^|*x_ij_*,*u_j_*) = var(*u_j_*) + var(*ε_ij_*), where var(*u_j_*) represents the variance between the clinic and the patient level var(*ε_ij_*) = π^2^/3. The municipality level was excluded in the multi-level structure and included as part of the random effect level because POCT of HbA1c is organized by GPs at the clinics rather than the municipalities. All patient level markers were included as co-variates and continuous co-variates were divided into intervals. Age was grouped into 10 years age bands, cost co-variates were grouped into trisections, and the *Charlson Comorbidity Index* was grouped into three groups: 1 [0], 2 [1–7], and 3 [8–14]. Next, the estimated model (1) was used to calculate and report adjusted odds ratios (OR) that are conditional on the random effects. The Bayesian information criterion (BIC), Akaike information criterion (AIC), and the *Wald Chi2-Test* were used to compare a fully adjusted model including: (a) demographic markers, (b) socioeconomic markers, (c) health utilization markers, (d) case mix markers, and (e) municipality type markers to a partly adjusted model including (a) and (b). The lowest AIC score and BIC score indicate the best model. A Wald test was used to test the overall significance of the model. The variance inflation factor (VIF) was used to measure the extent of multi-collinearity among covariates (VIF > 10). A measure of *R*-squared for logistic regression was used to estimate the explanatory power of the models [24]. The intra-class correlation coefficient was used to estimate the proportion of overall residual variability associated with the clinic level. However, no physician or clinic level data were included since this type of data was unavailable. All data and descriptive analyses were performed using the statistical program Stata Version 14 (Stat/IC, College Station, TX, USA) at Statistics Denmark. Participant consent and ethics approval was not required to conduct this study due to the Danish Act on Processing of Personal Data.

## 3. Results

Our desk research revealed that the Capital Region of Denmark is the only region which has allowed GPs to use the incentive for POCT of patients. Among the 44,981 patients with diabetes in the Capital Region, 14,660 patients were listed in 201 clinics which provided POCT of HbA1c and 6349 patients actually received POCT during the year in 2011.

### 3.1. Variation in POCT of HbA1c at Patient Level

Table 1 shows descriptive patient characteristics of the 6349 patients with diabetes who received POCT of HbA1c versus the 38,632 patients who did not receive POCT of HbA1c in the Capital Region in 2011.

Table 1 shows that 14.1% of patients with diabetes received POCT of HbA1c (one or more). There were significant differences between patients who received POCT versus non-POCT patients. A significantly larger proportion of patients monitored via POCT were male, had a short education, and had another ethnicity than Danish. In regards to the age distribution, the mean age of POCT patients was approximately three years greater than non POCT patients and there were more POCT patients in the older age intervals and fewer in the younger intervals than among non-POCT patients. POCT patients had a lower mean income, a larger proportion were retired, and a smaller proportion were unemployed. These results indicate that POCT is more frequently used for a subset of patients with diabetes.

On average, patients in the POCT group received 2.38 POCTs in 2011 and were characterized by a significantly lower consumption of health care services in general—in terms of hospital ACSCs, standard hospital visits, and emergency admissions. On average, patients in the POCT group experienced fewer preventable hospital admissions (ACSC) (0.025 per patient) compared to patients in the non POCT group (0.039 per patient). This trend was similar in the subgroup of diabetes-related ACSCs, where the average number of admissions in the POCT group was only 0.004 admissions per patient versus 0.012 per patient in the non POCT group according to the ACSC-algorithm. POCT patients also experienced a significantly lower morbidity burden in terms of the Charlson Comorbidity index (based on hospital activity), drug costs, inpatient DRG-costs, outpatient hospital cost, and total health care cost. In contrast to this general trend, POCT patients experienced a significantly higher cost in general practice (mean = €362.8 vs. 250.0) and other primary care areas (mean = €410.7 vs. 395.8). This is related to a higher number of GP visits. The proportion of patients who received POCT was lower in peripheral municipalities, higher in intermediate municipalities, and similar in urban municipalities. A significantly higher proportion of POCT patients, with a value of 41% vs. 34.7% of non-POCT patients, were tested using a blood sample for laboratory testing at the GP clinic. This was also the case within POCT clinics, where 41% of POCT patients and 26.4% of non POCT patients had a blood sample taken. Fewer POCT patients used walk-in laboratories. The coefficient of variation (CV) for the continuous variables in Table 1 shows the level of variability between patients and allows for a comparison of variability between the markers. One difference between the markers is that there is a relatively high variation in health care utilization and cost among the patients in the hospital sector. For instance, the CV is higher for the different elements of hospital utilisations, hospital costs, and the Charlson Comorbidity index than for health care utilization in primary care for both POCT and non-POCT clinics. Other differences in the level of variability between patients in POCT clinics and non-POCT clinics are ACSCs, hospital visits, and ED visits where the variability is lower in POCT clinics.

### 3.2. Variation in POCT of HbA1c at Clinic Level

Table 2 shows descriptive clinic characteristics for the 201 clinics using POCT with 14,660 patients versus the remaining non-POCT clinics with 30,321 patients in the Capital Region.

There were significant variations in the reported clinic characteristics (aggregated patient data) across POCT versus non POCT clinics in the Capital Region—in terms of list size, number of patients with diabetes per clinic, and number of patients with diabetes per 1000 patients. The difference in the clinic’s proportion of patients by municipality type was not statistically significant. The average prevalence of POCT in all clinics was 11.6%, within a range of 0–85%. Across the two subgroups of clinics, the distributions of the list size of clinics, prevalence of POCT at the clinic level, number of patients with diabetes per clinic, and number of patient with diabetes per 1000 patients were different. In POCT clinics, the mean use of POCT was 42.6%. POCT clinics were significantly larger than non POCT clinics—in terms of the number of listed patients, the number of patients with diabetes, and the number of patients with diabetes per 1000 listed patients. The majority of both POCT (90.7%) and non POCT patients (94.0%) were located in urban municipalities. The rest of the clinics were located in intermediate (8.8% vs. 3.8%) and peripheral municipalities (0.5% vs. 2.2%).

### 3.3. Logit Analyses of Variation in POCT of HbA1c at Patient Levelg

Table 3 shows odds ratios for POCT based on patient characteristics among patients with diabetes in POCT clinics for two models. Model 1 comprises age, gender and, socioeconomic markers, while Model 2 shows the results for the fully adjusted model. Below, we focus on Model 2 because it was seen to be the best model.

Overall, Model 2 in Table 3 shows that POCT was associated with patient characteristics among patients with access to POCT of HbA1c in their clinics. The estimated proxy for explanatory power (*R*-squared) in Model 2 indicates that the included patient characteristics were able to explain about 37% of the variation in POCT of HbA1c among the 14,660 patients who were linked to the 201 clinics using POCT in the Capital Region. The OR for use of POCT is higher for older age groups, than the youngest age group (reference group). But the age groups with the older age have relatively lower OR values than the middle aged groups. Males have higher odds than females; furthermore, patients with a low education (only compulsory public school) and other ethnicity have higher odds than the reference groups. In contrast, patients who were single had a 9% decrease in the odds for the use of POCT vs non-singles.

In general, measures of morbidity burden and need in terms of case mix measures based on data from the hospital sector reveal that the OR for the use of POCT is 53% lower among patients who have a Charlson Comorbidity index in the range (1–7) and 76% lower among patients with a Charlson Comorbidity index in the range (8–14) versus patients with an index of 0. In Table 3, this trend towards lower OR can also be seen in patients who experienced higher outpatient hospital costs. In contrast, patients who experienced a high morbidity burden in terms of GP cost (second and third trisection) and other parts of primary care, have higher OR values for POCT versus patients in the first trisection of cost. Finally, patients with high drug costs (third trisection) also appear to have significantly lower OR values for POCT vs. patients with low drug costs (first trisection). This shows that POCT patients are more likely to be male, have a short education, other ethnicity, and experience high GP, low outpatient, and relatively low drug costs compared to other patients with diabetes. The estimated intra-class correlation coefficient indicates that 22.5% of the overall residual variability is associated with variability at the clinic level. Thus, the majority of the unexplained variation in POCT of HbA1c among patients with diabetes seems to be unobserved patient characteristics, such as preferences for POCT of HbA1c. The AIC and BIC scores confirm that the fully adjusted model (Model 2) is the best model. The VIF did not reveal multicollinearity above standard thresholds (VIF < 10).

## 4. Discussion

The results of the study show that POCT testing varies considerably within general practice in Denmark. Surprisingly, only one Danish region has implemented POCT of HbA1c, despite the national framework agreement. Within the Capital Region where the fee was implemented, this study reveals remarkable variation in the use of POCT.

### 4.1. Patient Level

A comprehensive set of patient characteristics were able to explain a significant part of the variation in POCT. Both the group difference test results of patients with diabetes who received POCT versus non POCT patients in Table 1 and the results in Table 3, show that POCT patients are different from non POCT patients. Males with a low education level and ethnicity other than Danish have significantly higher odds for POCT. The fact that these patients receive more POCT may reflect patient preferences and/or GP preferences to offer POCT to these types of patients [25]. High patient costs in general practice and other parts of primary care also indicate higher odds for POCT. In comparison, high patient costs for drugs and/or morbidity in terms of the Charlson Comorbidity index (the latter based on hospital data) mean lower odds for POCT. These results reveal that general practice primarily monitors patients with a low Charlson Comorbidity index and low consumption of drugs. Since patient characteristics, such as case mix markers, may be perceived as proxies for patient needs, it can be argued that there is a link between use of POCT and patient needs. However, this study does not include patient preferences. To the best of our knowledge, relatively little has been published regarding the impact of POCT on patient preferences. One study indicates a high level of patient satisfaction with on-site POCT in a primary care setting [26]. The remaining variation at the patient level is related to factors such as omitted variables and unobserved variation at patient, clinic or the regional level.

Surprisingly, a higher proportion of POCT patients (41%) had a blood sample taken than non POCT patients (34.7%). The explanation could be that non POCT patients were either tested for HbA1c in hospital ambulatory clinics and walk-in laboratories or did not receive at least one annual test. Data on both the extent of walk-in laboratory testing of HbA1c and the patient group who did not receive any testing was not available for this study. More research is needed to explore the relationship between the three ways of Hba1c testing and the group of patients who may not receive any tests. Furthermore, the finding that POCT patients visit their GP more than Non POCT patients was unexpected. However, this study cannot rule out that POCT patients visit their GPs more than other patients, due to other provider and/or patient characteristics. For instance, patients who experience a lower morbidity burden may be more proactive and mobile, in addition to being more likely to seek out expert clinicians who are trained in special procedures and offer unique services such as POCT.

### 4.2. Clinic Level

Clinics which have applied POCT of HbA1c are larger and have a larger number of patients with diabetes per 1000 patients, indicating that clinics adopting POCT are more focused on diabetes management than non POCT clinics. The GPs in these clinics may be more engaged and/or specialized in diabetes management. Larger GP clinics or clinics with a large population of patients with diabetes may be able to make it more convenient and cost-efficient to invest in POCT equipment. For instance, clinics that have invested in POCT equipment may also have an economic incentive to perform more POCT in order to make their investment profitable in terms of production costs (direct labor costs and materials) and transaction costs (coordinating the production).

The special FFS fee for POCT in the Capital Region may cause the undersupply or oversupply of POCT in some clinics compared to other clinics. From one perspective, the incentive for POCT seems to be economically unattractive for GPs compared to the remuneration of standard laboratory testing. This may be the case for the underuse of POCT (or overuse of laboratory testing) by GPs motivated by extrinsic incentives. From another perspective, POCT may be perceived as attractive by other GPs who are interested in fewer consultations per patient with diabetes and are less motivated by FFS fees due to other reasons, such as intrinsic motivation or a large number of listed patients. However, it can be argued that the economic incentive for POCT of HbA1c in Danish general practice has not been strong enough to minimize variation in the use of and access to POCT. Further studies, which include provider characteristics at the clinic level as covariates, are warranted to explore the unexplained part of variation (ICC (Interclass correlation coefficient) = 0.225) at the clinic level.

### 4.3. Regional Level

The variation in the use of POCT at the inter-regional level can be explained by circumstances related to agreements. The incentive in the financial framework agreement from 2008 has not been implemented on a national level. Furthermore, the level of the politically negotiated fee for POCT may be too low compared to the alternative FFS structure for laboratory testing in order to subsequently change the practice of the majority of GPs. Unsuccessfully implementing the new national fee may contribute to variation in equal access to health care services. The differences in economic incentives to implement POCT and the related distribution of resources seem to be due to interregional dissimilarities in approaching diabetes management and regional administrative opinions about FFS incentives and/or the availability/prioritization of resources, which do not necessarily reflect patient needs and preferences. Therefore, it does not appear likely that the present delivery of POCT is optimal. Why should the technology be optimal in one region, but not in other regions? Proponents may argue that POCT may be preferred if testing features of the technology are equivalent to those in central laboratories. Furthermore, POCT may significantly improve the timeline to better control clinical follow-up in remote locations [27]. Opponents may argue that the use of POCT may be inappropriate if the testing features of the technology are not equal to the quality in central laboratories. Furthermore, according to the implementation of the agreement in the capital region, the use of the new FFS incentive has been restricted in use. The incentive is only intended to facilitate a more timely control of existing patients with diabetes. The GPs are not allowed to apply the new FFS incentive for diabetes screening among patients with diabetes symptoms.

Previous literature does not provide clear evidence that POCT in general practice always improves the quality of care and cost efficiency of care. However, when utilized properly, a part of the literature argues that POCT has been shown to yield measurable improvements in patient care, workflow efficiency, and even provide significant financial benefits [7]. For instance, the proper use of POCT requires that there is a focus on the local organization of tasks regarding sampling, analytical work, and in particular, quality assurance of equipment and procedures [28]. The quality of tests must be so high and the coordination so good in primary care that the measurements can match the laboratory service that the physicians receive at hospitals [29,30]. However, the literature does not provide strong evidence that POCT improves patient health outcomes in general practice, has a comparable analytical quality to laboratory testing, is cost-effective compared to usual care, or that patients and health professionals find POCT satisfactory [31,32]. Nevertheless, the literature shows that the cost of early and effective management of diabetes (e.g., via frequent screening and management using POCT) is more than compensated for by the savings in avoiding long term complications [33]. Thus, we consider it possible that the lower (or higher) use of POCT might be a cause of higher (or lower) comorbidity and a greater (reduced) need for drug treatment.

The inability of the authorities to cover the GPs’ work with standardized rules, guidelines, and the co-existence of several sets of alternative fees for the testing of HbA1c in diabetes management in general practice seems to stimulate variation in the way in which HbA1c testing is performed. An approach to remedy the variation could be to eliminate one of these alternative ways of remunerating testing of HbA1c. Theory about making or buying decisions such as transaction cost analysis provides a decision structure for choosing among laboratory testing (make) within the regions’ public organization or POCT in private GP clinics (buy), if the testing features of the two technologies are assumed to be equal [34]. Differences in the culture around blood sampling at the regional and clinic level may also contribute to the described variation in POCT. For instance, some regions such as the Central Region of Jutland collected blood samples free of charge in 2011, while other regions were asking GPs to pay for transporting samples to the central laboratories.

## 5. Limitations

The application of the POCT fee among patients linked to clinics is only the best available proxy for the extent of POCT of HbA1c in primary care. How accurate the register data is at reflecting reality is unknown without validation. The lack of a link between the use of POCT and the individual GP due to the nature of the Health Service Register is also a limitation. A link would have allowed us to analyse variation at the GP level rather than the clinic level.

Opponents can argue that the quality of registering in the Danish Health Service Register may be inappropriate, despite the fact that GPs have an explicit economic incentive to register this activity. Fluctuations in the quality of the registered utilization data could be due to several circumstances: Some GPs may not apply the incentive according to the agreement and some GPs may be incentivized to oversupply services remunerated by FFS. Other GPs may apply POCT equipment without charging for it. Implementers of POCT may assume that the testing features of the POCT technology are equivalent to those employed in central laboratories. However, research and discussions about the appropriateness of POCT of HbA1c in diabetes management reflect that this is a controversial assumption [7,28,30,35]. In one region, a hearing among senior staff at the regional laboratories and a special advisory diabetes committee resulted in the decision not to implement POCT due to concerns about the quality of POCT. Finally, this study did not include a proxy measure for the severity of diabetes. In future research, it may be relevant to explore whether proxy measures of severity such as time since diagnosis or treatment type have influenced the POCT of HbA1c. Despite these limitations, we find that a better understanding about variation in the use of health services for patients with diabetes is a relevant tool to place warranted and unwarranted variation in the mind-set of decision makers and may contribute to the improved organization of diabetes management.

## 6. Conclusions

This study demonstrates variation in the use of POCT of HbA1c in Danish general practice related to patient characteristics, GP clinic random effects, and variation in negotiated agreements at the regional level. Despite a national framework agreement regarding a POCT of HbA1c fee, the fee has only been implemented in one out of five Danish regions. In the Capital Region, where the POCT fee has been implemented, a significant part of the described variation in the use of POCT can be explained by observed patient characteristics. The POCT of HbA1c seems to be used more on a specific subgroup of patients with diabetes in terms of demographic markers, socioeconomic markers, morbidity burden case mix markers and costs. POCT patients are more likely to be of male gender, have a higher age, a short education, other ethnicity, and experience high GP, low outpatient, and relatively low drug costs compared to other patients with diabetes. Clinics using POCT of HbA1c are larger and have a larger number of patients with diabetes per 1000 patients. It appears relevant to reassess the system for POCT of HbA1c in general practice across the five Danish regions to secure equal access to health services for GPs, as well as patients. Further studies are warranted in order to assess the impacts of POCT of HbA1c on health care outcomes.

## Figures and Tables

**Table 1:** Descriptive patient characteristics for POCT vs. non POCT in the Capital Region 2011.

Patient Characteristics	All	POCT			Non POCT			Test of Differences *
Variables	Mean	Mean	SD	CV	Mean	SD	CV	(*p*-value)
N	44,981	6349	-	-	38,632	-	-	-
**Demographic markers**								
Age (years)	64.1	66.7	11.3	0.17	63.7	14.0	0.22	<0.001 ^B^
**Distribution based on age (%)**								
20–39	5.7	1.5	-	-	6.4	-	-	<0.001 ^A^
40–49	9.0	5.8	-	-	9.5	-	-	<0.001 ^A^
50–59	17.7	17.3	-	-	17.8	-	-	0.226 ^A^
60–69	31.0	33.9	-	-	30.5	-	-	<0.001 ^A^
70–79	24.4	29.1	-	-	23.7	-	-	<0.001 ^A^
80–89	10.7	11.4	-	-	10.6	-	-	0.031 ^A^
90–	1.45	1.06	-	-	1.5	-	-	0.003 ^A^
Gender (male = 1) (%)	53.5	54.5	49.6	0.91	53.4	49.7	0.93	<0.011 ^A^
**Socioeconomic markers**								
Income (€ per patient):	29,875	29,224	25,717.1	0.88	29,982	31,231	0.96	0.003 ^B^
Unemployed (%)	5.3	4.3	20.2	4.69	5.4	22,572	4.18	0.011 ^A^
Short education (%)	35.5	38.0	48.3	1.27	35.1	47.7	1.36	<0.001 ^A^
Other ethnicity (%)	17.2	18.7	38.9	2.08	17.0	37.6	2.21	<0.001 ^A^
Single (%)	37.4	36.5	48.2	1.32	37.5	48.4	1.29	0.189 ^A^
Retired (%)	64.1	70.5	45.8	0.65	63.1	48.6	0.77	<0.001 ^A^
**Health care utilisation**								
ACSC diabetes (# per patient)	0.011	0.004	0.07	17.51	0.012	0.15	12.51	<0.001 ^B^
Total ACSCs (# per patient)	0.037	0.025	0.22	8.80	0.039	0.29	7.33	<0.001 ^B^
Hospital visits (# per patient)	0.48	0.38	1.04	2.73	0.50	1.24	2.47	<0.001 ^B^
ED visits (# per patient)	0.16	0.14	0.46	3.31	0.17	0.54	3.20	<0.001 ^B^
Number of GP visits (# per patient)	7.04	8.61	6.20	0.72	6.79	6.11	0.90	<0.001 ^B^
Number of POCT of HbA1c (# per patient)	0.34	2.38	1.52	0.64	0	-	-	<0.001 ^B^
**Case mix markers**								
Charlson Comorbidity index	0.92	0.58	1.15	1.98	0.97	1.37	1.41	<0.001 ^B^
Cost of all GPs (€ per patient)	265.9	362.8	199.5	0.55	250.0	202.5	0.81	<0.001 ^B^
Other primary care costs (€ per patient)	397.9	410.7	455.9	1.11	395.8	530.4	1.34	0.007 ^B^
Drug costs (€ per patient)	695.1	660.6	713.4	1.08	700.7	819.8	1.17	<0.001 ^B^
Inpatient hospital DRG-costs (€ per patient)	2360.9	1813.3	6292.2	3.47	2451.0	8161.8	3.33	<0.001 ^B^
Outpatient hospital DAGS-costs (€ per patient)	1789.9	1259.2	3450.2	2.74	1877.1	5180.8	2.76	<0.001 ^B^
Total health care cost including drugs (€ per patient)	5560.8	4557.6	8067.0	1.77	5725.6	10,935.9	1.91	<0.001 ^B^
**Municipality type—rural district classification**								
Peripheral municipalities (%)	4.7	1.5	-	-	5.3	-	-	0.552 ^A^
Rural municipalities (%)	0	0	-	-	0	-	-	-
Intermediate municipalities (%)	7.4	11.1	-	-	6.8	-	-	0.014 ^A^
Urban municipalities (%)	87.9	87.3	-	-	88.0	-	-	0.113 ^A^
Walk-in laboratory in municipality (%)	43.0	40.0	48.8	1.22	43.4	49.5	1.14	0.236 ^A^
**Testing of HbA1c**								
Point of care testing D7403—one or more (%)	14.1	100	0	0	0	0		-
Blood sample for central lab—one or more (%)	35.6	41.0	49.2	1.20	34.7	47.5	1.37	<0.001 ^A^

Coefficient of variation (CV) = SD/Mean. * Test of differences between proportions adjusted for clustering by general practices. ^A^ Mixed effects logistic regression, ^B^ Mixed effects generalized linear model.

**Table 2:** Descriptive clinic variations in POCT of HbA1c in the Capital Region in 2011.

Clinic Characteristics	All	POCT Clinic	Non POCT Clinic	Test of Difference (*p*)
Total number of clinics	746	201	545	
**Proportion of patients by municipality type (%)**				
Peripheral municipalities	1.7	0.5	2.2	0.169 ^A^
Rural municipalities	0	0	0	-
Intermediate municipalities	5.2	8.8	3.8	0.078 ^A^
Urban municipalities	93.1	90.7	94.0	0.310 ^A^
**List size of GP clinic (# patients)**				0.001 ^B^
Mean	1580.3	1810.4	1495.5	
P5	754	794	752	
P95	3474	3,935	3204	
**Prevalence of POCT at clinic level (%)**				<0.001 ^B^
Mean (%)	11.6	42.6		
P5 (%)	0	12.2		
P95 (%)	58.2	70.6		
**Number of patients with diabetes per clinic**				<0.001 ^B^
Mean	59.7	72.9	54.8	
P5	20	22	19	
P95	143	159	135	
Range				
**Number of patients with diabetes per 1000 patients**				0.002 ^B^
Mean	38.0	40.8	36.9	
P5	17.2	12.2	16.7	
P95	64.7	70.6	64.2	

^A^ Test of differences between proportions (chi-square). ^B^ Test of distribution difference (Mann-Whitney).

**Table 3:** Odds ratios and POCT of HbA1c of diabetic patients in 2011.

Patient Characteristics	Odds Ratio	*p*	95% Conf. Interval	Odds Ratio	*p*	95% Conf. Interval	Reference Group
	Model 1			Model 2			
**Demographic Makers**							
Age 40–49	2.99	<0.001	(2.30, 3.88)	2.40	<0.001	(1.78, 3.23)	Age 20–39
Age 50–59	5.58	<0.001	(4.38, 7.13)	3.90	<0.001	(2.96, 5.13)	Age 20–39
Age 60–69	7.57	<0.001	(5.91, 9.70)	4.57	<0.001	(3.45, 6.04)	Age 20–39
Age 70–79	8.81	<0.001	(6.80, 11.40)	4.69	<0.001	(3.51, 6.28)	Age 20–39
Age 80–89	7.49	<0.001	(5.71, 9.84)	3.30	<0.001	(2.42, 4.48)	Age 20–39
Age 90–	4.14	<0.001	(2.76, 6.22)	1.67	0.026	(1.06, 2.61)	Age 20–39
Gender (male = 1)	1.12	0.003	(1.04, 1.21)	1.35	0.001	(1.23, 1.48)	Female = 0
**Socioeconomic Markers**							
Income (€) 2nd trisection	0.91	0.068	(0.83, 1.01)	0.92	0.122	(0.82, 1.02)	1st trisection
Income (€) 3rd trisection	0.92	0.130	(0.82, 1.02)	0.99	0.994	(0.88, 1.13)	1st trisection
Unemployed	1.14	0.164	(0.95, 1.38)	0.97	0.800	(0.78, 1.21)	Not unemployed
Short education	1.17	<0.001	(1.08, 1.26)	1.15	0.003	(1.05, 1.26)	Not short education
Other ethnicity	1.37	<0.001	(1.23, 1.53)	1.25	<0.001	(1.10, 1.41)	Danish
Single	0.90	0.010	(0.83, 0.97)	0.91	0.041	(0.83, 0.99)	Not single
Retired	1.03	0.628	(0.92, 1.15)	0.89	0.097	(0.78, 1.21)	Not retired
**Case Mix Markers**							
Charlson Comorbidity index (1, 7)				0.47	<0.001	(0.42, 0.52)	Index = 0
Charlson Comorbidity index (8, 14)				0.24	0.002	(0.09, 0.61)	Index = 0
GP costs (€) 2nd trisection				6.81	<0.001	(6.09, 7.61)	1st trisection
GP costs (€) 3rd trisection				14.29	<0.001	(12.59, 16.22)	1st trisection
Other primary costs (€) 2nd trisection				1.46	<0.001	(1.30, 1.64)	1st trisection
Other primary costs (€) 3rd trisection				1.38	<0.001	(1.23, 1.55)	1st trisection
Total cost of drugs (€) 2nd trisection				0.95	0.319	(0.85, 1.05)	1st trisection
Total cost of drugs (€) 3rd trisection				0.87	0.015	(0.78, 0.97)	1st trisection
Outpatient costs (€) 2nd trisection				0.72	<0.001	(0.64, 0.81)	1st trisection
Outpatient costs (€) 3rd trisection				0.48	<0.001	(0.43, 0.56)	1st trisection
**Municipality Type Markers**							
Intermediate municipality				0.45	0.380	(0.077, 2.65)	Peripheral
Rural municipality				0.39	0.298	(0.67, 2.24)	Peripheral
Walk-in laboratory in municipality				1.04	0.638	(0.88, 1.23)	No walk-in lab
Constant	0.10	<0.001	(0.08, 0.13)	0.14	0.031	(0.02, 0.83)	
Number of patients (N)	14,660			14,660			
Number of groups	201			201			
R^2^ (Tjurs’ coef. of discrimination)	0.17			0.37			
Wald Chi2 (df)	(14) 578.05 *p* < 0.0001			(27) 2743.52 *p* < 0.0001			
AIC	17,766.56			14,349.12			
BIC	17,888.04			14,569.31			
Variance inflation factor (mean)	2.35			2.54			
Intraclass correlation coefficient	0.175		(0.144, 0.211)	0.225		(0.187, 0.268)	

## Abbreviations

POCT	Point-of-care-testing
PLO	General Practitioners Organsiation
GP	General Practitioner
ACSC	Ambulatory Care Sensitive Condition
DRG	Diagnosis related groups
DAGS	Danish Ambulatory Grouping System
VIF	Variance Inflation factor
ICC	Interclass correlation coefficient
